# Extrahepatic biliary tract visualization using near-infrared fluorescence imaging with indocyanine green: optimization of dose and dosing time

**DOI:** 10.1007/s00464-020-08058-6

**Published:** 2020-10-07

**Authors:** Qiangxing Chen, Rou Zhou, Jiefeng Weng, Yueyuan Lai, Hui Liu, Jiao Kuang, Shuai Zhang, Zhaofeng Wu, Wen Wang, Weili Gu

**Affiliations:** 1 Department of Surgery, Guangzhou First People’s Hospital, School of Medicine, South China University of Technology, No. 1 Panfu Road, Yuexiu District, Guangzhou, 510180 Guangdong China; 2 Department of Endocrine, Guangzhou First People’s Hospital, School of Medicine, South China University of Technology, No. 1 Panfu Road, Yuexiu District, Guangzhou, 510180 Guangdong China

**Keywords:** Fluorescence cholangiography, Indocyanine green, ICG, Near-infrared, Laparoscopic cholecystectomy

## Abstract

**Background:**

The dose and dosing time of indocyanine green (ICG) vary among fluorescence cholangiography (FC) studies. The purpose of this prospective, randomized, exploratory clinical trial was to optimize the dose and dosing time of ICG.

**Methods:**

PubMed was searched to determine the optimal dose. To optimize the dosing time of ICG, a clinical trial was designed with two parts. The first part included patients with T tubes for more than 1 month. After the patient was injected with ICG, bile was collected at 10 time points to explore the change and trends of bile fluorescence intensity (FI). In addition, the results of the first experiment were used to setup a randomized controlled trial (RCT) that aimed to find the optimal dosing timing for ICG injections for laparoscopic cholecystectomy (LC). During surgery, imaging data were collected for analysis.

**Results:**

After performing a systematic review, the ICG injection dose for each patient in the clinical trial was 10 mg. Five patients were included in the first part of the study. Bile collected 8 h after ICG injection had a higher FI than bile collected at other time points (*p* < 0.05), and the FI of bile collected 20 h after ICG injection was nearly zero. In the second part of the experiment, 4 groups of patients (6 patients per group) were injected with 10 mg ICG at 8, 10, 12 and 14 h prior to surgery. The distribution of bile duct FI (*p* = 0.001), liver FI (*p* < 0.001), and common bile duct (CBD)-to-liver contrast (*p* = 0.001) were not the same in each group. Further analysis with the Bonferroni method revealed the following: (1) the FI of the CBD in the 8 h group was significantly different from that in the 14 h group (adjusted *p* < 0.001); (2) the liver FI of the 8 h group was higher than that of the 10 h group (adjusted *p* = 0.042) and the 14 h group (adjusted *p* < 0.001); and (3) the CBD-to-liver contrast of the 8 h group was lower than that of the 10 h group (adjusted *p* = 0.013) and the 14 h group (adjusted *p* = 0.001).

**Conclusion:**

ICG FC enables the real-time identification of extrahepatic bile ducts. The optimal effect of FC can be achieved by performing 10 mg ICG injections 10 to 12 h prior to surgery.

**Electronic supplementary material:**

The online version of this article (10.1007/s00464-020-08058-6) contains supplementary material, which is available to authorized users.

Laparoscopic cholecystectomy (LC) is one of the most commonly performed surgical procedures [1, 2], with more than 60,000 operations performed in Japan and approximately 750,000 in the United States every year [3]. The most serious complication is bile duct injury (BDI), with an incidence of 0.3–1.5% [4–6]. BDI has a significant impact on quality of life and survival. To decrease the risk of BDI, extra intraoperative visualization techniques, such as intraoperative ultrasound and cholangiogram (IOC), have been introduced [7]. However, these technologies are not widely used in the clinic due to their limitations.

Over the last several years, the intraoperative visualization of bile ducts using near-infrared (NIR) light and the fluorescent dye indocyanine green (ICG) were introduced during cholecystectomy [3, 8, 9]. ICG is an intravenously delivered agent that is almost exclusively metabolized by hepatic parenchymal cells and secreted into the bile [10–12]. After intravenous injection, ICG concentrates in the bile and emits light with a peak wavelength at approximately 830 nm when stimulated by NIR light (700–900 nm) [10, 11]. This technique provides real-time fluorescent visualization of vascular and biliary structures even before the dissection of Calot’s triangle [3, 13], whereas IOC is generally performed after the dissection of the cystic duct (CD) [14]. A randomized clinical trial showed that NIR fluorescence cholangiography (FC) was significantly better than white light alone in visualizing extrahepatic biliary structures during LC [15].

There is no doubt that high-quality fluorescence imaging can help surgeons identify the anatomy of the extrahepatic biliary tract. The dose and dosing time of ICG are key factors that affect the performance of high-quality fluorescence imaging because if fluorescence imaging is performed directly after the administration of ICG, the liver will be highly fluorescent while bile ducts will not yet contain enough ICG (Fig. 1A). In contrast, if fluorescence imaging is performed too long after the administration of ICG, the fluorescence intensity (FI) of the common bile duct (CBD) and liver will be so low that it cannot be conducive to identifying anatomical structures (Fig. 1B). The optimal signal would involve a high fluorescence signal in bile ducts and a low fluorescence signal from the liver tissue in the background (Fig. 1C). However, among the published studies, the dosage and timing of ICG administration widely varied [16]; for instance, various studies used 2.5 mg administered within 0.5 h or 1 h prior to surgery [3, 8, 17], 12.5 mg administered within 0.5 h prior to surgery [13], and 0.05 mg/kg administered within 1 h prior to surgery [18–20]. However, only a few studies have tried to optimize the dose and timing of administration [16, 21–23].

**Fig. 1:**
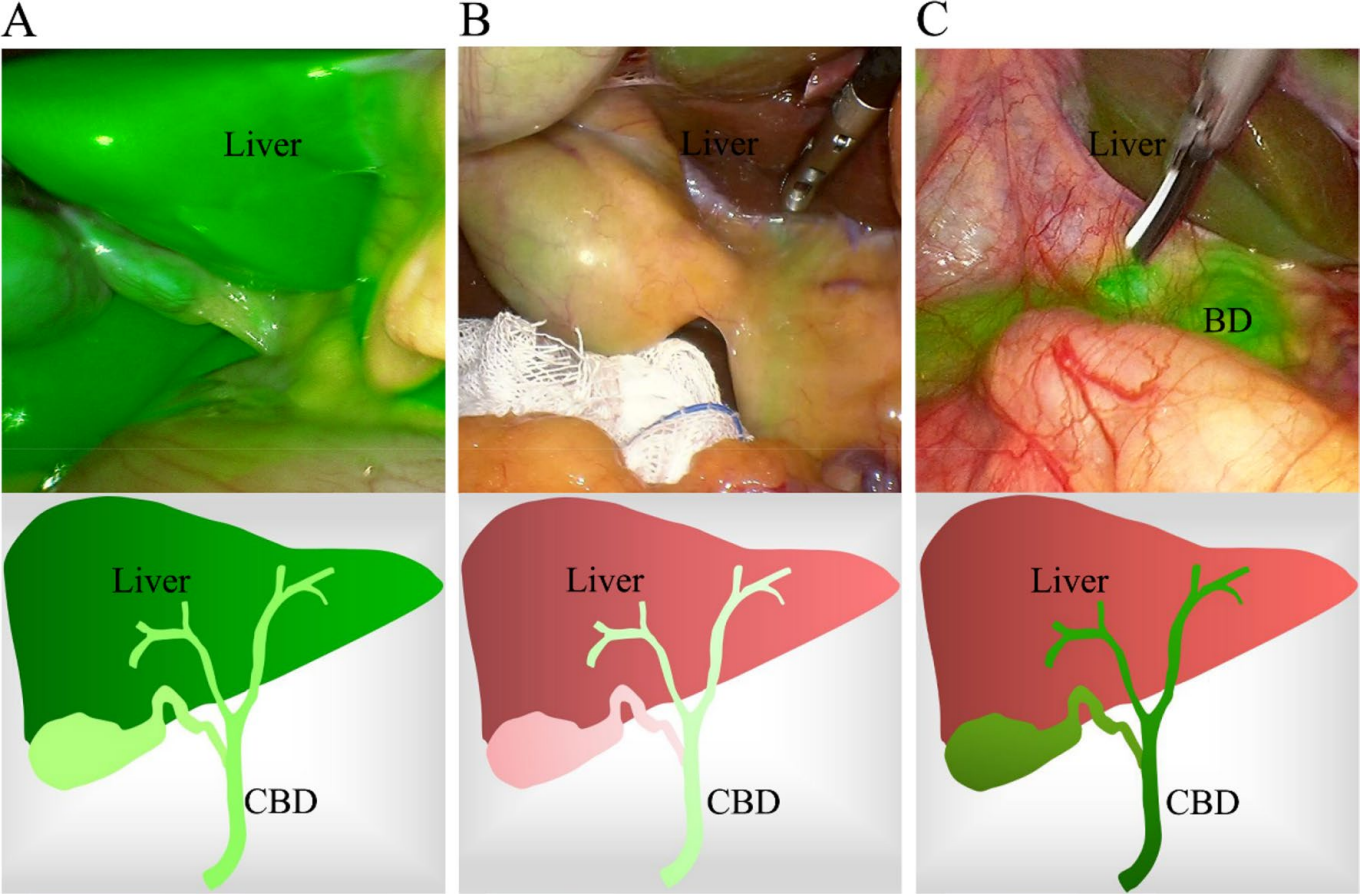
Effect of ICG injection dose or dosing time on FC. **A** The liver is highly fluorescent, while bile ducts did not yet contain enough ICG to locate the biliary tract. **B** Both the FI of the CBD and liver were so low that they could not be used to identify anatomical structures. **C** Bile ducts were highly fluorescent, while liver tissue in the background exhibited a low fluorescence signal

Therefore, the purpose of this study was to investigate the optimal ICG injection dose and dosing time in laparoscopic FC.

## Materials and methods

### Determination of the ICG injection dose

The optimal ICG injection dose was confirmed by systematically searching PubMed. In PubMed, we searched the literature using the search term “((indocyanine green[Title/Abstract]) OR ICG[Title/Abstract]) AND cholangiography[Title/Abstract].” The search results were filtered as follows. First, articles written in English and describing 3 or more patients were selected. Subsequently, the title and abstract of selected articles were independently reviewed by two researchers.

### Intraoperative NIR fluorescence imaging system

**Fig. 2:**
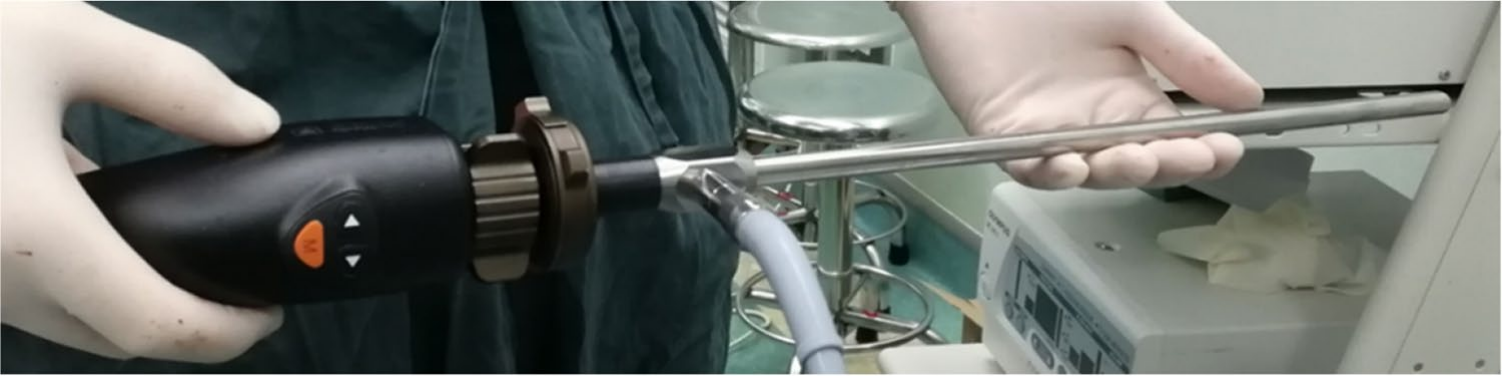
The NIRF imaging system used for this study was developed by OptoMedic (GuangZhou, China)

**Fig. 3:**
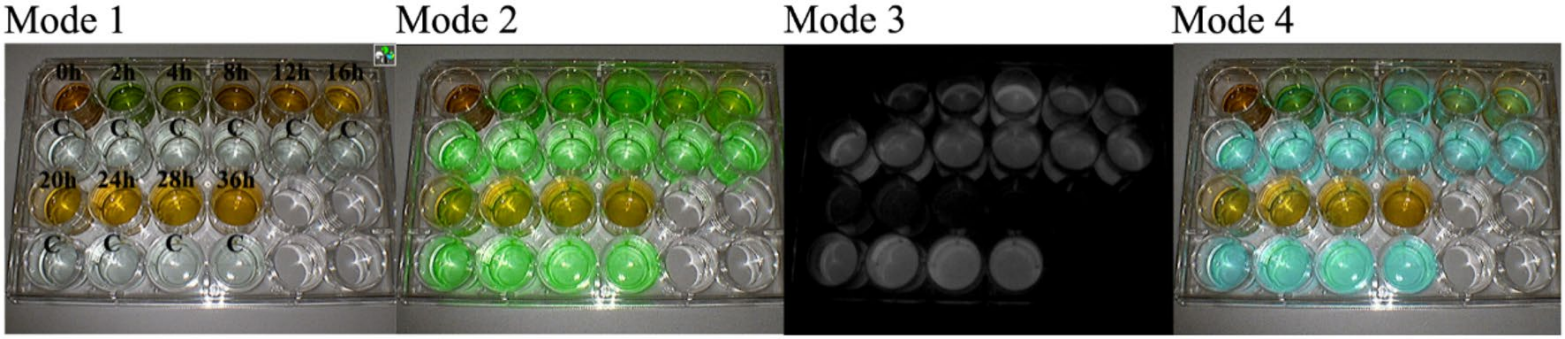
FI changes of bile collected from patients with T tubes at different time points after ICG injection using the NIRF imaging system. 0 h, bile collected from T tube before ICG injection; 2 h, 4 h, 8 h, 12 h, 16 h, 20 h, 24 h, 28 h, and 36 h, bile collected from T tubes 2, 4, 8, 12, 16, 20, 24, 28, and 36 h after ICG injection, respectively; C, control fluids containing both ICG and albumin (albumin concentration: 0.01 g/mL; ICG concentration: 0.01 mg/mL)

Laparoscopic imaging was performed using a high-definition fluorescence laparoscope (OptoMedic Endoscopes, China) through a standard 12 mm subumbilical trocar port. The system was equipped with a visible and NIR light source (Fig. 2). In addition, the NIR fluorescence (NIRF) imaging system could switch between 4 different modes, which included modes 1, 2, 3, and 4: mode 1, white light imaging alone; mode 2, green fluorescence imaging with white light imaging; mode 3, original fluorescence imaging without white light imaging; and mode 4, fluorescence and white light imaging after pseudo-color processing (Figs. 3 and 6).

### Clinical trial

The protocol was approved by the medical institutional review board of our hospital. All patients or their family members provided written informed consent before participation in the study. This study was registered in the Chinese Clinical Trial Registry (ChiCTR) (www.chictr.org.cn), which was accepted by the International Committee of Medical Journal Editors (No. ChiCTR1900028137). To investigate the optimal ICG injection dose and dosing time for laparoscopic FC, we divided the clinical trial into two parts.

#### Part one

##### Patients

Five participants were invited to participate in this part of the study starting November 1, 2019. The enrollment criteria included the following: (1) patients with indwelling T tubes for more than 1 month after extrahepatic biliary tract surgery; (2) patients without biliary obstruction; (3) imaging examinations, such as computed tomography (CT) or ultrasound, did not detect cirrhosis, and the serum bilirubin was less than 30 µmol/L; and (4) age ≥ 18 years. The exclusion criterion was patients with a history of adverse reactions or known allergies to ICG, iodine, or iodine dyes.

##### Study design

This part of the study used a repeated measure design. Bile was collected from the T tube before ICG injection. Then, the optimal dose of ICG was injected at 7 pm, and bile was collected from the T tube at 9 time points after ICG injection. The 9 time points for collecting bile were 2 h, 4 h, 8 h, 12 h, 16 h, 20 h, 24 h, 28 h and 36 h after ICG injection. Bile collected at the above 10 time points was added to the respective experimental wells, and a solution containing both ICG and albumin was added to the control wells (albumin concentration: 0.01 g/mL; ICG concentration: 0.01 mg/mL). Subsequently, a NIRF imaging system was used to process the specimens and acquire fluorescent images. Finally, the FI of each well in the fluorescent images was measured by Image-Pro Plus (IPP; produced by Media Cybernetics Corporation, USA). To acquire the FI ratio, the FI of the experimental wells and control wells were compared.

#### Part two

##### Patients

Patients were included after the study results of part one were analyzed. The inclusion criteria were as follows: (1) patients had indications for extrahepatic biliary tract surgery and could be operated on using laparoscopy; (2) serum bilirubin was less than 30 µmol/L; (3) imaging examinations, such as CT or ultrasound, did not detect cirrhosis; and (4) age ≥ 18 years. Patients with a history of adverse reactions or known allergies to ICG, iodine, or iodine dyes were excluded.

##### Study design and sample size

This prospective, randomized, multi-arm, exploratory clinical trial was conducted at the Surgery Department of Guangzhou First People's Hospital (Guangzhou, China). We expected that a certain number of patients could be enrolled during a short time for statistical analysis. As the needed sample size was small, we decided to use a nonparametric test (the Kruskal–Wallis *H* test) for analysis. Both Dwivedi [25] and Mundry [26] pointed out that 24 observations are needed for the Kruskal–Wallis *H* test with four groups. Therefore, the total sample size of this part of the study was 24, and 6 patients were included in each of the four groups.

##### Randomization

Twenty-four eligible patients were randomly divided into 4 groups (groups A, B, C and D; allocation ratio 1:1:1:1) using simple randomization. A randomization sequence was created using SPSS 19.0 statistical software, and the set seed was 20191101. Whether a patient was assigned to group A, B, C or D was determined by the statistician based on the randomization sequence. The grouping information was unknown to the patients, ICG injection staff, fluorescence image acquisition staff, and FI testing staff. After a patient was accepted by the recruitment team and before the ICG injection, the appropriate numbered envelope was opened by the statistician at the office. The card inside showed whether the patient assigned to group A, B, C or D, the dose and dosing time of the ICG injection for the patient, and the statistician informed only the ICG injection staff about the dose and timing of the ICG injection. After the fluorescence images were collected, only the FI testing staff participated in the measurement of FI. The FI testing staff were blinded to the photos they assessed. The number of each fluorescence image corresponds to the random number of each patient and the number of the fluorescence image does not involve grouping information. Finally, the above results were summarized and analyzed by the statistician.

##### Interventions

Each included patient was injected with the optimal dose of ICG via the peripheral vein at 4 time points that were determined by analyzing the study results of part one. Before the dissection of the Calot’s triangle, a NIRF imaging system was used to observe the extrahepatic bile duct and surrounding liver tissues, and the images were saved at the same time.

##### Measurements of FI

The FI of the CBD and liver was measured using IPP software. The FI ratio of the CBD/liver (CBD-to-liver contrast) was calculated.

### Statistical analysis

We examined whether all data obtained in this study were normally distributed. Data are presented as the mean ± standard deviation (SD) or median according to the data distribution. A two-sided *p*-value of < 0.05 was considered statistically significant. For between-group analyses, comparisons of continuous variables were carried out by analysis of variance (ANOVA) or by the Kruskal–Wallis *H* test. The Bonferroni method was used for pairwise comparisons between multiple groups to correct the significance level. The statistical analysis of the data was performed by statisticians using SPSS software 19.0 (SPSS Inc., Chicago, Illinois, USA). Power analysis was not performed prior to beginning the study, as similar studies have not been performed. A post hoc power analysis was conducted utilizing the statistical software program GPower (version 3.1.9.2 Dusseldorf, Germany).

#### Follow up

Patients were closely observed after ICG injection, and if any side effects occurred, they were recorded and promptly treated.

## Results

### The optimal injection dose of ICG

Until December 18, 2019, a total of 76 articles were identified. After reviewing all titles and contents of the abstracts, 29 eligible articles were selected (Supplementary table). Most studies (14/29) used 2.5 mg ICG administered within 2 h prior to fluorescence imaging. Four studies (4/29) used an ICG dose of 0.05 mg/kg within 1 h prior to surgery. The timing of ICG administration differed from 0 (intraoperatively) up to 24 h. The majority of studies (23/29) performed ICG injection within 60 min prior to surgery. Only four studies (4/29) investigated FC using different doses of ICG and times of administration (Table 1). Based on the analysis of the results of the 4 studies, a better bile duct-to-liver contrast was obtained with an ICG injection dose of 10 mg before surgery, and this ideal contrast can be maintained for a longer period of time.

**Table 1:** Articles about optimizing dose and dosing time of ICG injection in laparoscopic fluorescence cholangiography

Study	Year	Dose of ICG (mg)	Timing of injection	Bile duct-to-liver ratios (BLR)^a^
Verbeek et al. [21]	2014	5, 10, 20 mg	5 or 10 mg/30 min before incision; 10 or 20 mg/24 h prior to surgery	1. BLR < 1, 5 mg or 10 mg/0.5 h 2. BLR = 2.3 (range 1.1–6.2), 10 mg/24 h 3. BLR = 1.7 (range 1.6–2.9), 20 mg/24 h
Zarrinpar et al. [22]	2016	0.02, 0.04, 0.08, and 0.25 mg/kg	10 ± 3 min, 45 ± 15 min, and 3 ± 1 h prior to surgery	1. BLR < 1, 0.02, 0.04, 0.08, and 0.25 mg/kg 2. BLR < 1, 10 ± 3 min, 45 ± 15 min, and 3 ± 1 h
Boogerd et al. [16]	2017	5 or 10 mg	5 mg/0.5, 2, 4, or 6 h prior to surgery; 10 mg/4, 6, or 24 h prior to surgery	1. BLR > 1, 5 mg/3 to 7 h 2. BLR > 1, 10 mg/5 to 25 h^b^
Tsutsui et al. [23]	2018	25 mg	0 h, 3 h, 6 h, 9 h, 12 h, 15 h, 18 h, 24 h prior to surgery	1. BLR > 1, 25 mg/15, 18, and 24 h

^a^Bile duct-to-liver ratios (BLR), the ratio of fluorescence intensity between the bile duct and liver

^b^BLR > 1, 10 mg/5 to 25 h, Bile duct-to-liver ratios were greater than 1 after 10 mg ICG injection 5 to 25 h prior to surgery

### Changes in bile FI after ICG injection in patients with T tubes

#### Patient characteristics

Patient characteristics are shown in Table 2. There were 2 males and 3 females, and the age ranged from 51 to 74 years. All 5 patients had T tube indwelling due to CBD exploration, and the T tube indwelling times were 34 days, 35 days, 41 days, 42 days, and 50 days. CT or ultrasound did not detect liver cirrhosis in these 5 patients. Biliary cholangiography was performed before T tube extubation, and the results showed that the 5 patients had no biliary obstruction. Liver function tests showed that the total bilirubin value of 5 patients ranged from 5.6 to 23.5 µmol/L.

**Table 2:** Patients characteristics of part one

No. of patients	5
Age, years, mean ± SD	64 ± 10
Sex(male/female)	2/3
Diagnose	
Common bile duct stone	5
Total Bilirubin (µmol/L), mean ± SD	12.2 ± 6.8
Time to indwell T tube (day), mean ± SD	42.4 ± 5.4
Cholangiography	
Unobstructed	5
Obstruction	0
Evaluation of cirrhosis using CT or ultrasound	
Suggestive of cirrhosis	0
Suggestive without cirrhosis	5

#### FI of collected bile

**Fig. 4:**
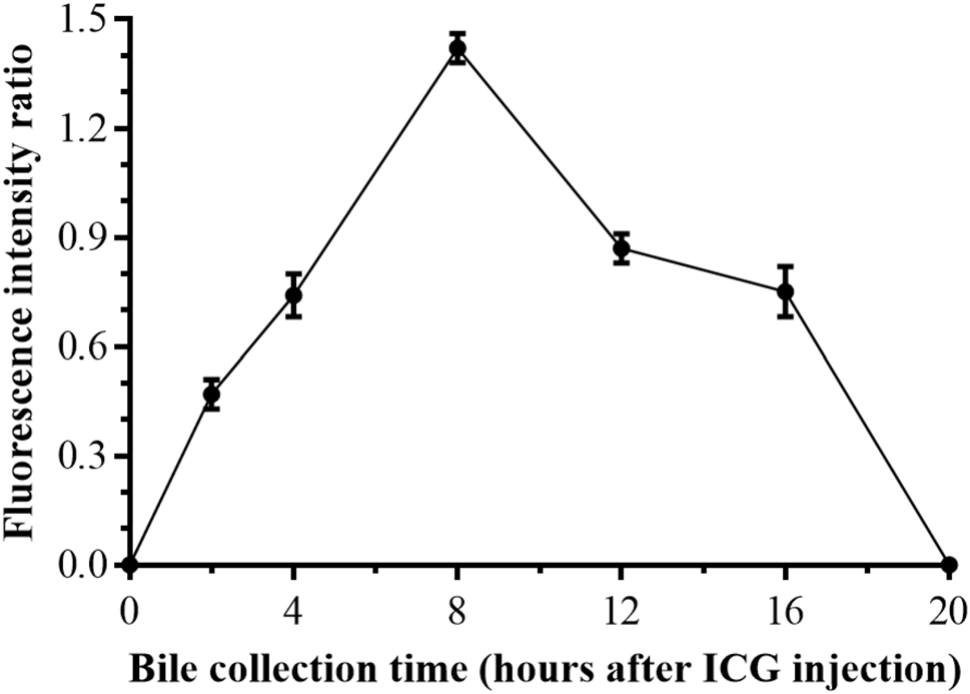
Trend of changes in the FI ratio

Enrolled patients were injected with 10 mg ICG at 7 pm, and bile was collected from the T tube 2 h, 4 h, 8 h, 12 h, 16 h, 20 h, 24 h, 28 h, and 36 h after ICG injection. No patient experienced side effects because of the ICG injection. Collected bile and control fluids were added to a 24-well plate and processed with a NIRF imaging system to obtain fluorescent images (Fig. 3). Subsequently, IPP software was used to measure the FI of the liquid in each well, and the ratio of FI between experimental wells and the control wells was calculated. Figure 4 shows the trend of the change in the FI ratio. The statistical method of one-way repeated measures ANOVA can be used to calculate the effect of time on the FI ratio. The results of the Shapiro–Wilk test showed that the data of each group were normally distributed (*p* > 0.05). The result of Mauchly’s sphericity test showed that the assumption that the covariance matrix was spherical could not be rejected (*χ*^2^ = 14.282, *p* = 0.19). The sample means and SDs of the FI ratio at 5 time points, which included 2 h, 4 h, 8 h, 12 h and 16 h after ICG injection, were (0.47 ± 0.04), (0.74 ± 0.06), (1.42 ± 0.04), (0.87 ± 0.04), and (0.75 ± 0.07), respectively. The results showed that the difference among the 5 time points was significant (*F* = 348.126, *p* < 0.001). The FI ratio at 8 h after ICG injection was 0.96 (95% confidence interval (CI) 0.78–1.13) higher than that at 2 h after ICG injection (*p* < 0.001) and 0.69 (95% CI 0.48–0.89) higher than that at 4 h after ICG injection (*p* < 0.001). The FI ratio at 12 h after ICG injection was 0.55 (95% CI 0.40–0.70) less than that at 8 h after ICG injection (*p* < 0.001). The FI ratio at 16 h after ICG injection was 0.67 (95% CI 0.46–0.89) lower than that at 8 h after ICG injection (*p* = 0.001). Post hoc analysis demonstrated that the sample sizes used were sufficient to detect an effect size of 0.989 at approximately 100% power and alpha = 0.05.

### FC after injecting ICG at different time points before surgery

#### Patient characteristics

**Fig. 5:**
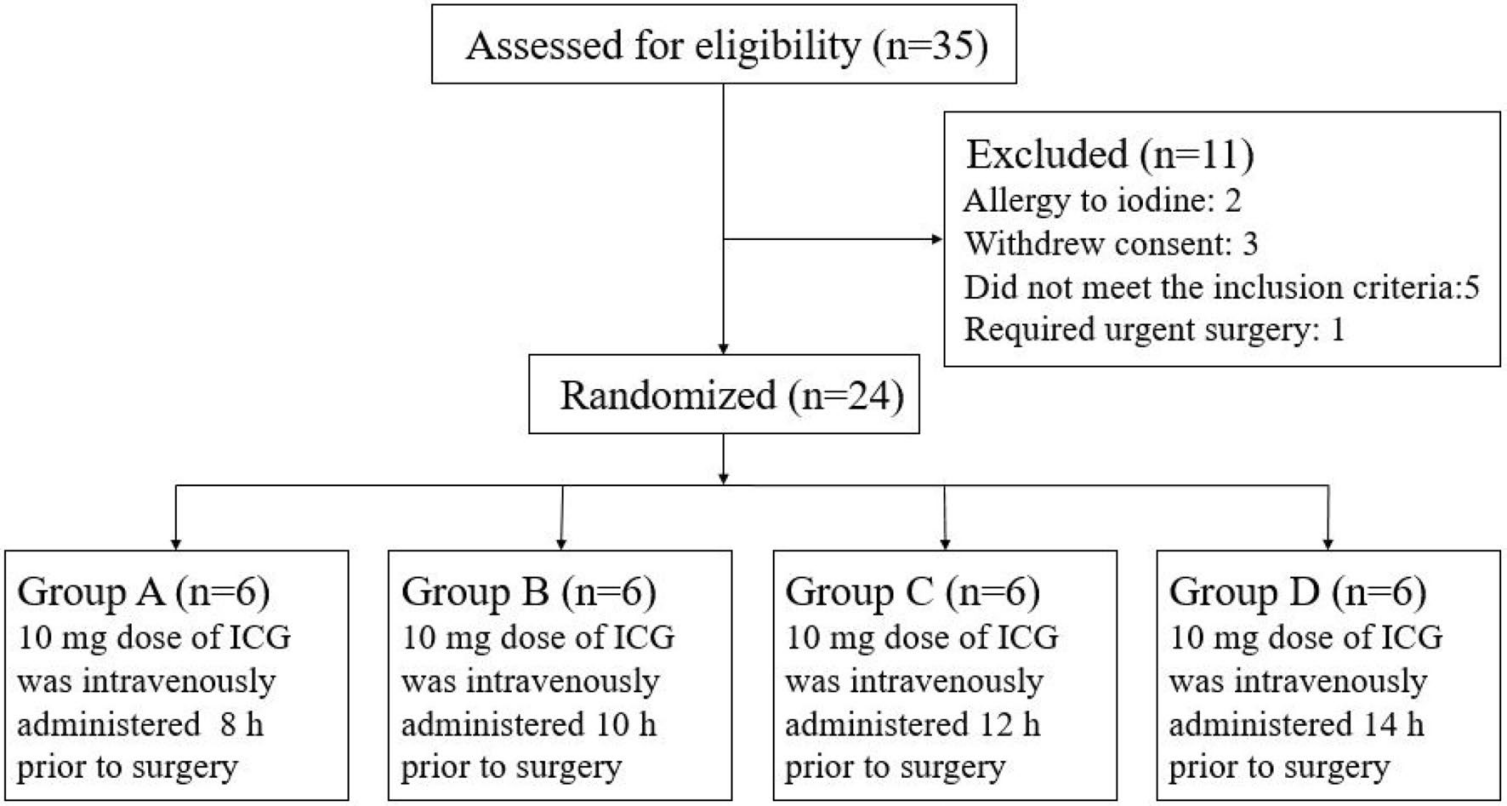
CONSORT diagram showing the flow of participants through each stage of the trial

From November 15, 2019 to December 15, 2019, we included 24 eligible patients. The Consolidated Standards of Reporting Trials (CONSORT) diagram shows the flow of participants through each stage of the study (Fig. 5). Thirty-five patients were assessed for eligibility, and 24 were randomized. The characteristics of the included patients are shown in Table 3; the study included 13 males and 11 females aged 24 to 84 years. Eighteen patients were diagnosed with gallstones with chronic cholecystitis, 2 patients were diagnosed with gallstones with acute cholecystitis, 1 patient was diagnosed with choledocholithiasis with gallstones, 2 patients were diagnosed with gallbladder polyps, and 1 patient was diagnosed with gallbladder adenomatoid hyperplasia. Liver function tests showed that the total bilirubin value of the 24 patients ranged from 10.2 to 24.7 µmol/L.

**Table 3:** Patients characteristics of part two

No. of patients	24	Diagnose	
Age, years		Gallstones with chronic cholecystitis	A/5^a^, B/6, C/4, D/3
Group A	53 ± 15	Gallstones with acute cholecystitis	A/1, B/0, C/0, D/1
Group B	58 ± 18	Gallbladder polyp	A/0, B/0, C/1, D/1
Group C	64 ± 13	Choledocholithiasis with gallstones	A/0, B/0, C/0, D/1
Group D	55 ± 17	Gallbladder adenomatoid hyperplasia	A/0, B/0, C/1, D/0
Sex (male/female)		Operative procedures	
Group A	2/4	Laparoscopic cholecystectomy (LC)	23
Group B	2/4	LC with CBD exploration	1
Group C	5/1	Gallbladder wall thickness	
Group D	4/2	*d* ≤ 3 mm	A/1, B/4, C/4, D/4
Total bilirubin (µmol/L)		3 mm < *d* < 6 mm	A/3, B/2, C/2, D/1
Group A	15.6 ± 3.3	*d* ≥ 6 mm	A/2, B/0, C/0, D/1
Group B	17.6 ± 4.3	Evaluation of cirrhosis using CT or ultrasound	
Group C	19.1 ± 7.4	Suggestive of cirrhosis	A/0, B/0, C/0, D/0
Group D	18.4 ± 6.7	Suggestive without cirrhosis	A/6, B/6, C/6, D/6

^a^A/5, there are 5 patients diagnosed gallstones with chronic cholecystitis

#### FI of the extrahepatic biliary tract and liver

**Fig. 6:**
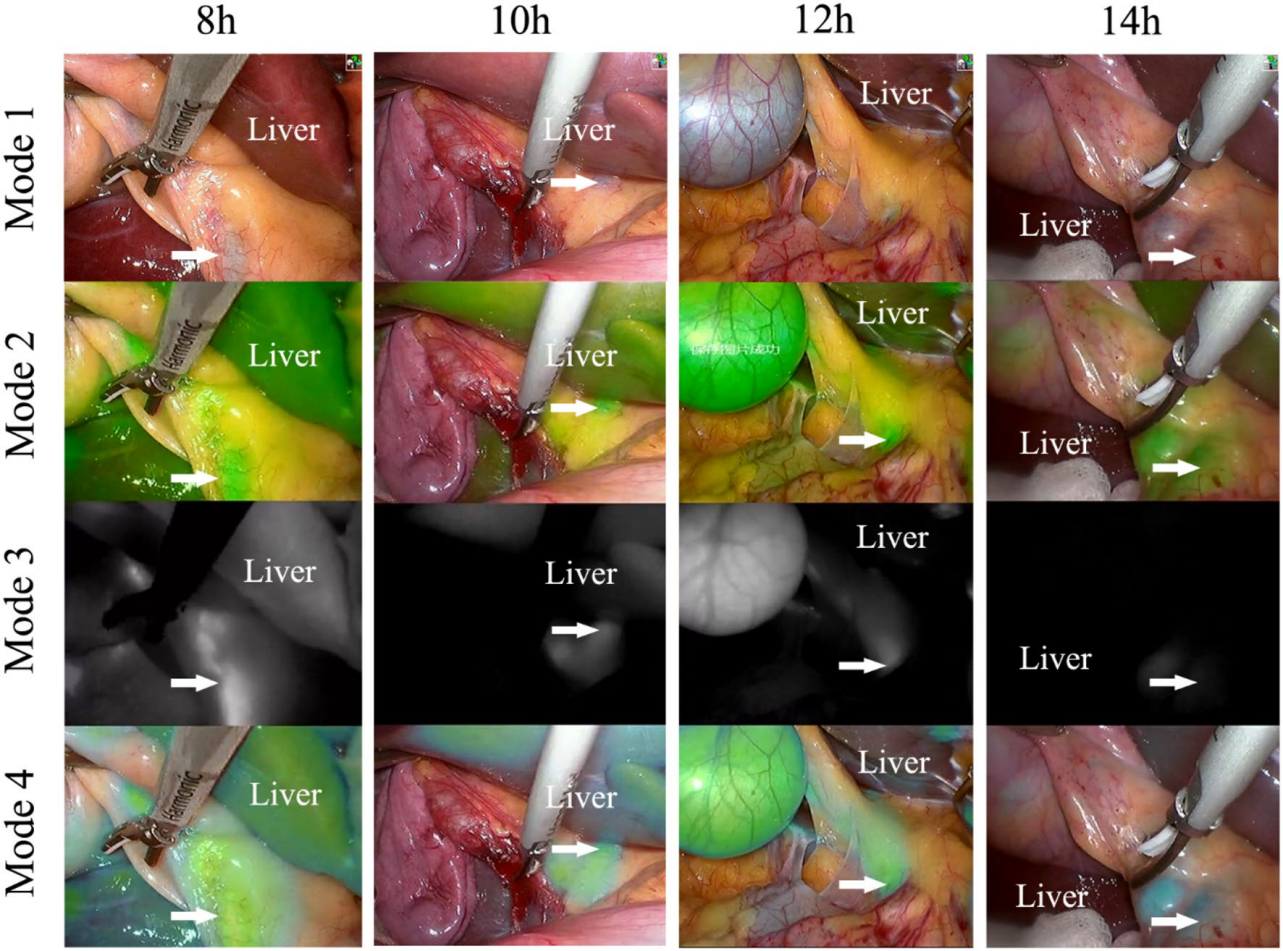
The fluorescence of the liver and extrahepatic biliary tract (arrow) of the four groups of patients after using the NIRF imaging system during the operation. 8 h, patients in group A were injected with 10 mg ICG 8 h prior to surgery. 10 h, patients in group B were injected with 10 mg ICG 10 h prior to surgery. 12 h, patients in group C were injected with 10 mg ICG 12 h prior to surgery. 14 h, patients in group D were injected with 10 mg ICG 14 h prior to surgery

After analyzing the results of part one, the ICG injection time points for patients in groups A, B, C, and D were 8 h, 10 h, 12 h and 14 h prior to surgery, respectively. Each included patient was intravenously injected with 10 mg of ICG**.** No patients experienced side effects because of the ICG injection. Subsequently, the fluorescence of the liver and extrahepatic biliary tract of patients could be seen after using the NIRF imaging system during the operation (Fig. 6, see Video, [Video. Techniques of FC during LC, 2 min 46 s, 125 MB]). Based on the images obtained during the operation, a preliminary conclusion was made that the FI of the liver in the 8 h group was stronger than that in the 10 h, 12 h, and 14 h groups, and the FI of the bile duct in the 14 h group was lower than that in the 8 h, 10 h, and 12 h groups (Fig. 6).

**Fig. 7:**
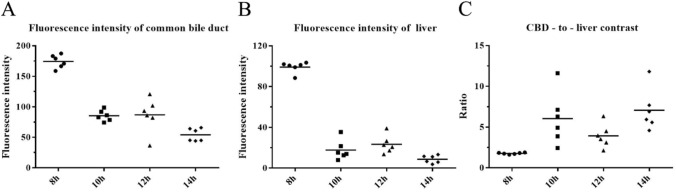
Evaluation of the effect of 10 mg ICG injected at 4 time points (8, 10, 12 and 14 h prior to surgery). **A** The FI of the CBD in the 8 h group was significantly different from that in the 14 h group (Bonferroni method, adjusted *p* < 0.001). **B** The liver FI of the 8 h group was higher than that of the 10 h group (Bonferroni method, adjusted *p* = 0.042) and the 14 h group (Bonferroni method, adjusted *p* < 0.001). **C** The CBD-to-liver contrast of the 8 h group was lower than that of the 10 h group (Bonferroni method, adjusted *p* = 0.013) and the 14 h group (Bonferroni method, adjusted *p* = 0.001)

Scatter plots (median) were used to evaluate the effect of 10 mg ICG injected at 4 time points, including 8, 10, 12 and 14 h prior to surgery (Fig. 7). The indicators evaluated include the FI of the CBD (Fig. 7A), the FI of the liver (Fig. 7B), and the FI ratio of the CBD/liver (CBD-to-liver contrast) (Fig. 7C).

The statistical data of the second part were not normally distributed (Shapiro–Wilk test, *p* < 0.05). The differences in the distribution of bile duct FI, liver FI and CBD-to-liver contrast between different groups were compared using the Kruskal–Wallis *H* test. (1) The distribution of bile duct FI was not the same in each group, and the differences were statistically significant (*H* = 17.467, *p* = 001). The average ranks of bile duct FI in groups A, B, C, and D were 21.5, 11.83, 12.17, and 4.5, respectively. A post hoc comparison using the Bonferroni method to correct the significance level showed that the difference in the distribution of bile duct FI between groups A and D (adjusted* p* < 0.001) was statistically significant, and the differences between the other groups was not statistically significant. (2) The distribution of liver FI was not the same in each group, and the differences were statistically significant (*H* = 18.747, *p* < 001). The average ranks of bile duct FI in groups A, B, C, and D were 21.5, 10.5, 13.83, and 4.17, respectively. A post hoc comparison using the Bonferroni method to correct the significance level showed that the differences in the distribution of liver FI between groups A and B (adjusted *p* = 0.042) and groups A and D (adjusted *p* < 0.001) were statistically significant, and the difference between the other groups was not statistically significant. (3) The distribution of CBD-to-liver contrast was not the same in each group, and the differences were statistically significant (*H* = 16.087, *p* = 001). The average ranks of CBD-to-liver contrast in the four groups of A, B, C, and D were 3.5, 16.0, 11.67, and 18.83, respectively. A post hoc comparison using the Bonferroni method to correct the significance level found that the differences in the distribution of CBD-to-liver contrast between groups A and B (adjusted *p* = 0.013) and groups A and D (adjusted *p* = 0.001) were statistically significant, and the differences between the other groups was not statistically significant.

## Discussion

Fernando Dip et al. [15] reported that NIFC was significantly better than white light alone for visualizing extrahepatic biliary structures during LC. Moreover, large studies have reported that FC with ICG enables the real-time identification of extrahepatic bile ducts during surgery and is suggested to be safe [24]. However, the effectiveness of FC is affected by many factors, such as the dosage and timing of ICG administration and patient pathology. The optimal dosage of ICG and dosing time are particularly important for obtaining high-quality fluorescence imaging of bile ducts, yet only a few studies [16, 21–23] have tried to optimize the dose and timing of administration. Large studies focusing on patient outcomes should demonstrate whether fluorescence imaging reduces bile duct injuries.

Therefore, this study aimed to optimize the dose of ICG and dosing time during FC. In our systematic review, we determined that the ICG injection dose for patients included in this study was 10 mg. Subsequently, our research team creatively explored the trend in changes in FI of bile collected at different time points after ICG injection using a NIRF imaging system (Fig. 4). We found that bile collected 8 h after ICG injection had a higher FI than bile collected at other time points, while the FI of bile collected 20 h after ICG injection was nearly zero (Figs. 3 and 4). Based on the results of part one of the study, the following inferences can be made: (1) after ICG injection, the ICG concentration in the CBD gradually increases at first and then gradually decreases; (2) by injecting 10 mg of ICG 8 h before surgery, we are likely to observe a better fluorescence signal in the extrahepatic biliary tract during surgery; and (3) when 10 mg of ICG is injected 20 h prior to surgery, the extrahepatic biliary tract may not be observed with fluorescence during surgery. Therefore, our research team selected 4 time points (8 h, 10 h, 12 h, and 14 h before surgery) as the ICG injection time points for groups A, B, C, and D, respectively, in part two. The results showed that a satisfactory fluorescence image of the CBD can be obtained by injecting ICG at 8 h, 10 h and 12 h prior to surgery (Fig. 6). However, the CBD-to-liver contrast of the 8 h group was lower than that of the other three groups (Fig. 7). Although the CBD-to-liver contrast of the 14 h group was significantly higher than that of the 8 h group, the FI of the bile duct was too low to effectively distinguish the anatomy. Thus, a better visual effect with FC can be obtained by injecting 10 mg ICG 10 to 12 h before surgery.

The significant differences in the results of this experiment were derived from the following aspects of our study. (1) We creatively used bile collected from patients with T tubes for the first time to explore the trend in changes in bile FI after ICG injection. The experimental results accurately guided the ICG injection time in part two, thereby reducing the number of groups. (2) In the process of recruiting patients, we excluded patients who had major factors affecting ICG excretion so that the distribution of FI data would not be too discrete. The main factors affecting ICG excretion are hepatic blood flow, hepatocellular function, and unobstructed bile ducts. In fact, ICG and bilirubin bind to the same carrier in the transport process in hepatocytes and therefore exhibit competitive inhibition [27–29]. It should be noted that the limitations of this trial are the small sample size and the small number of patients with acute cholecystitis. Further studies with a larger sample size may be needed to verify the optimal dose and dosing time of ICG injection.

## Conclusions

FC enables the real-time identification of extrahepatic bile ducts. Furthermore, this study provides guidance on the timing and dosing of ICG. The optimal effect of FC can be achieved by performing 10 mg ICG injections 10 h to 12 h prior to surgery.

## Electronic supplementary material

Below is the link to the electronic supplementary material.

Supplementary file 1 Video. Techniques of FC during the dissection of Calot’s triangle in LC. The patient was 52-year-old man with gallbladder adenomatoid hyperplasia. He was injected with 10 mg ICG via the peripheral vein 10 h prior to surgery. Before LC, his serum bilirubin was 20.3 µmol/L. The operative time was 95 min, and blood loss was 15 mL. His postoperative course was uneventful (AVI 125112 kb)

Supplementary file 2 (DOCX 20 kb)

Supplementary file 3 (DOC 218 kb)

Supplementary file 4 (DOC 229 kb)

## Abbreviations

NIR: Near-infrared
ICG: Indocyanine green
LC: Laparoscopic cholecystectomy
NIRF: Near-infrared fluorescence
CBD: Common bile duct
BDI: Bile duct injury
IOC: Intraoperative cholangiogram
CD: Cystic duct
CT: Computed tomography
SD: Standard deviation
