# Supplementary material for: Extrahepatic biliary tract visualization using near-infrared fluorescence imaging with indocyanine green: optimization of dose and dosing time
Source: Surg Endosc. 2020 Oct 7;35(10):5573–82. doi: 10.1007/s00464-020-08058-6 (PMC8437885; doi:10.1007/s00464-020-08058-6)
Supplement: Supplementary file 2 — (DOCX 20 kb) [file 464_2020_8058_MOESM2_ESM.docx]

**Supplementary table: eligible articles about indocyanine green fluorescent cholangiography.**

| **No** | **Title** | **IF/author/Year** | **Objective or Conclusion** | **Dose and dosing time of ICG** |
| --- | --- | --- | --- | --- |
| 1 | Fluorescent Cholangiography in Laparoscopic Cholecystectomy: An Updated Canadian Experience. | 1.47  Bleszynski MS  2019 | administration of ICG enhances visualization of the biliary system during outpatient LC. | **Dose:** 1.6 mL of a 25 mg/10 mL solution; **dosing time:** 0nce the patient underwent general anesthesia and endotracheal tube placement. |
| 2 | True single‑port cholecystectomy with ICG cholangiography through a single 15‑mm trocar using the new surgical platform “symphonX”: first human case study with a commercially available device | 3.209  Datta RR  2019 | Laparoscopic cholecystectomy in patients with cholecystitis and cholecystolithiasis using the symphonX platform through only one 15-mm trocar is feasible, safe, and more cost-efficient compared to robotic platforms. | **Dose:** using intravenous 5 mg ICG  **Dosing time:** 3 h before surgery. |
| 3 | Routine near infra-red indocyanine green fluorescent cholangiography versus intraoperative cholangiography during laparoscopic cholecystectomy: a case-matched comparison. | 3.209  Quaresima S  2019 | to evaluate safety and efficacy of near infra-red (NIR) indocyanine green (ICG) fluorescence structural imaging | **Dose:**Administered doses ranged from 3.5 to 13.5 mg  **Dosing time:** at diferent time intervals prior to the operation, |
| 4 | The role of indocyanine green fluoroscopy for intraoperative bile duct visualization during laparoscopic cholecystectomy: an observational cohort study in 70 patients | /  Ambe PC  2019 | Laparoscopic cholecystectomy with real-time indocyanine green fluorescence cholangiography enables a better visualization and identification of biliary tree | **Dose:** 0.5 ml of ICG was given (25mg/10ml)via **Dosing time:** intravenous infusion one hour prior to surgery. |
| 5 | Randomized Trial of Near-infrared Incisionless  Fluorescent Cholangiography | 9.476  Dip F  2019 | NIFC was statistically superior to WL alone visualizing extrahepatic biliary structures during laparoscopic cholecystectomy. | **Dose:** (0.05mg/kg) intravenously  **Dosing time:** at least 45 minutes before surgery. |
| 6 | Evaluation of laparoscopic cholecystectomy using  indocyanine green cholangiography including  cholecystitis: A retrospective study | 2.48  Mathes A  2019 | We might have been able to perform LC with more confidence, and we had been able to identify CD or CBD with ICG cholangiography. | **Dose:** 2.5mg ICG  **Dosing time:** approximately 2 hours before surgery |
| 7 | Optimal timing of preoperative indocyanine green administration for fluorescent cholangiography during laparoscopic cholecystectomy using the PINPOINT® Endoscopic Fluorescence Imaging System. | /  Tsutsui N  2018 | investigated the optimal timing of indocyanine green administration to allow fluorescent cholangiography | **Dose:** 25 mg  **Dosing time:** The timing of indocyanine green administration was set immediately before surgery and at 3, 6, 9, 12, 15, 18, and 24 h before surgery. |
| 8 | Prospective Evaluation of Precision Multimodal Gallbladder Surgery Navigation: Virtual Reality, Near-infrared Fluorescence, and X-ray-based Intraoperative Cholangiography. | 9.476  Diana M  2017 | We aimed to prospectively evaluate NIR-C, VR-AR, and x-ray intraoperative cholangiography (IOC) during robotic cholecystectomy. | **Dose:** 0.1 to 0.4 mg/kg of ICG  **Dosing time:** approximately 45 to 60 minutes before the incision, |
| 9 | A Technique to Define Extrahepatic Biliary Anatomy Using Robotic Near-Infrared Fluorescent Cholangiography. | 2.686  Maker AV  2017 | The objective of the study was to evaluate if near-infrared fluorescent cholangiography (NIFC) using indocyanine green (ICG) may aid in safe dissection to obtain the critical view. | **Dose:** 2.5 mg ICG intravenously  **Dosing time:** at the time of intubatio |
| 10 | The Best Approach for Laparoscopic Fluorescence Cholangiography: Overview of the Literature and Optimization of Dose and Dosing Time. | 1.472  Boogerd LSF  2017 | We first systematically identified all strategies for fluorescence cholangiography. Second, we aimed to optimize the dose of ICG and dosing time in a prospective clinical trial. | **Dose and dosing time:** 5 mg/0.5, 2, 4, or 6 hours prior to surgery;  **Dose and dosing time:** 10 mg/4, 6, or 24 hours prior to surgery. |
| 11 | Near-infrared fluorescence cholangiography assisted laparoscopic cholecystectomy versus conventional laparoscopic cholecystectomy (FALCON trial): study protocol for a multicentre randomised controlled trial. | 2.376  van den Bos J  2016 | designed to assess the potential added value of the NIRF imaging technique during LC. | **Dose:** 2.5 mg of ICG will be given intravenously.  **Dosing time:** after the induction of anaesthesia, |
| 12 | Intraoperative Laparoscopic Near-Infrared Fluorescence Cholangiography to Facilitate Anatomical Identification: When to Give Indocyanine Green and How Much. | 1.472  Zarrinpar A  2016 | These results suggest that a dose of 0.25 mg/kg administered at least 45 minutes prior to visualization facilitates intraoperative anatomical identification. | **Dose:** 0.02, 0.04, 0.08, and 0.25 mg/kg.  **Dosing time:** 10 ± 3 min, 45 ± 15 min, and 3 ± 1 h prior to sugery |
| 13 | Accuracy of Near Infrared-Guided Surgery in Morbidly Obese Subjects Undergoing Laparoscopic Cholecystectomy. | 3.603  Dip F  2016 | Our objective was to evaluate and detect variances of fluorescence imaging in obese and non-obese patients. | indocyanine green (ICG) dose of 0.05 mg/kg wasgiven intravenously 1 h preceding surgery |
| 14 | Techniques of Fluorescence Cholangiography During Laparoscopic Cholecystectomy for Better Delineation of the Bile Duct Anatomy. | 1.87  Kono Y  2015 | To evaluate the clinical and technical factors affecting the ability of fluorescence cholangiography | 1 mL of ICG (2.5 mg/mL) is intravenously injected prior to the entry of the patient into the operation room |
| 15 | Routine use of fluorescent incisionless cholangiography as a new imaging modality during laparoscopic cholecystectomy. | 3.209  Dip F  2015 | IOIFC appears to be a feasible, low-cost, expeditious, useful, and effective imaging modality when performing LC. | dose of 0.05 mg/kg of ICG was administered intravenously one hour prior to surgery |
| 16 | Near-infrared fluorescent cholangiography facilitates identification of biliary anatomy during laparoscopic cholecystectomy. | 3.209  Osayi SN  2015 | Our aims were to assess the safety and efficacy of NIRF-C for identification of biliary anatomy during LC. | two and a half milligrams of ICG were injected intravenously, approximately 60 minutes prior to making a surgical incision. |
| 17 | Effectiveness of intraoperative cholangiography using indocyanine green (versus contrast fluid) for the correct assessment of extrahepatic bile ducts during day-case laparoscopic cholecystectomy. | 2.686  Prevot F  2014 | we studied the effectiveness of IOC-IG (vs IOC-CF) during day-case laparoscopic cholecystectomy. | After the induction of anesthesia, 0.5 mg/kg bodyweight of IG was infused into a peripheral vein |
| 18 | Indocyanine green (ICG) fluorescent cholangiography during robotic cholecystectomy: results of 184 consecutive cases in a single institution. | 1.47  Daskalaki D  2014 | The aim of this study is to evaluate the efficacy of ICG and the Da Vinci Fluorescence Imaging Vision System in real-time visualization of the biliary anatomy. | a dose of 2.5 mg of ICG 45 minutes prior to the beginning of the surgical procedure |
| 19 | Optimization of near-infrared fluorescence cholangiography for open and laparoscopic surgery. | 3.21  Verbeek FP  2014 | The aim of the current study was to optimize ICG dose and timing for NIR cholangiography using a quantitative intraoperative camera system during open hepatopancreatobiliary (HPB) surgery. | 5 or 10 mg/30 min before incision;  10 or 20 mg /24 h prior to surgery. |
| 20 | Real-time near-infrared fluorescent cholangiography could shorten operative time during robotic single-site cholecystectomy. | 3.21  Buchs NC  2013 | The study purpose is to report our experience with ICG RSSC and compare the outcomes to standard RSSC. | 2.5-mg dose of ICG was administered intravenously during patient preparation by anesthesia, approximately 30–45 min before incision. |
| 21 | Real-time near-infrared (NIR) fluorescent cholangiography in single-site robotic cholecystectomy (SSRC): a single-institutional prospective study. | 3.209  Spinoglio G  2013 | This study aims to evaluate the efficacy and safety of this technique during single-site robotic cholecystectomy (SSRC). | During patient preparation, 2.5 mg of ICG was injected intravenously. |
| 22 | Fluorescence cholangiography during laparoscopic cholecystectomy: a feasibility study on early biliary tract delineation. | 3.209  Schols RM  2013 | study was designed to assess the feasibility and image quality of intermittent NIRFC during LC | One milliliter of ICG (2.5 mg/ml) intravenous injection of ICG directly after induction of anesthesia. |
| 23 | Indocyanine green reinjection technique for use in fluorescent angiography concomitant with cholangiography during laparoscopic cholecystectomy. | 1.345  Kaneko J  2012 | Fluorescent angiography using this ICG reinjection technique might enhance the safety of laparoscopic cholecystectomy. | ICG(1 mL, 0.05 mg/kg; Diagnogreen; Daiichi Sankyo, Tokyo,Japan) was injected intravenously 15 minutes before surgery  after dissection of the fluorescing cystic duct as a landmark.ICG (1 mL, 0.05 mg/kg) was reinjected intravenously. |
| 24 | Intra-operative fluorescent cholangiography using indocyanin green during robotic single site cholecystectomy. | 1.63  Buchs NC  2012 | the surgeon is able to assess the biliary anatomy by a non-invasive and non-ionizing method. This paper presents the first report of ICG imaging during a RSSC. | They underwent RSSC approximately 45 minutes after intravenous administration of ICG (2.5 mg). |
| 25 | Application of fluorescent cholangiography to single-incision laparoscopic cholecystectomy. | 3.209  Ishizawa T  2011 | Such properties of fluorescent cholangiography are expected to be helpful for ensuring the safety of SILC and expanding the indications for the procedure. | 1 ml of ICG (2.5 mg) was administered by intravenous injection after endotracheal intubation of the patient in the operating room. |
| 26 | Fluorescent cholangiography illuminating the biliary tree during laparoscopic cholecystectomy. | 5.586  Garden OJ  2010 | The aim of this study was to evaluate the ability of fluorescent cholangiography to detect anatomy of the biliary tree during LC as a possible substitute for conventional radiographic IOC | 2.5 mg ICG was injected intravenously 30 min before the patient entered the operating room or following intubation. |
| 27 | Intraoperative fluorescent imaging using indocyanine green for liver mapping and cholangiography. | 2.353  Aoki T  2010 | Application of this technique allows intraoperative identification of anatomical landmark in hepatobiliary surgery. | 5 ml of ICG was administered intravenously just before operation |
| 28 | Intraoperative exploration of biliary anatomy using fluorescence imaging of indocyanine green in experimental and clinical cholecystectomies. | 2.353  Tagaya N  2010 | evaluated the usefulness of intraoperative exploration of the biliary anatomy using fluorescence imaging with indocyanine green (ICG) in experimental and clinical cholecystectomies. | ICG (1.0 ml/body of 2.5 mg/ml ICG) was infused 1-2 h before surgery. |
| 29 | Intraoperative fluorescent cholangiography using indocyanine green: a biliary road map for safe surgery. | 4.45  Ishizawa T  2009 | We consider that fluorescent IOC using ICG is a safe and valuable procedure that provides a road map of the biliary tract anatomy in real time for safe hepatobiliary surgery | 1 mL of ICG (2.5 mg/mL) was injected IV 1 hour before the operation |
