# Supplementary figures and images for: Extrahepatic biliary tract visualization using near-infrared fluorescence imaging with indocyanine green: optimization of dose and dosing time
Source: Surg Endosc. 2020 Oct 7;35(10):5573–82. doi: 10.1007/s00464-020-08058-6 (PMC8437885; doi:10.1007/s00464-020-08058-6)

**
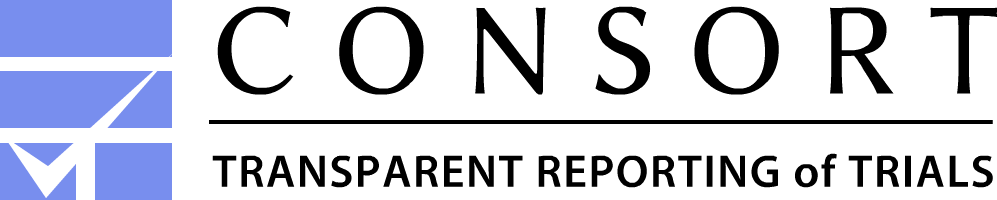
**

**CONSORT 2010 Flow Diagram**

**Enrollment**


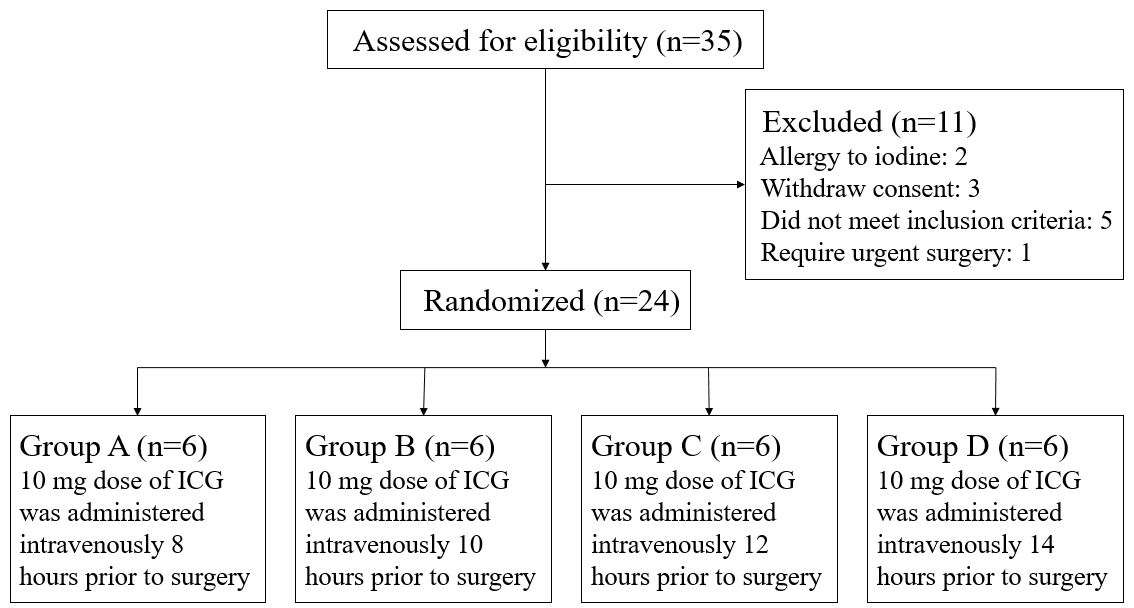

Supplement: Supplementary file 4 — (DOC 229 kb) [file 464_2020_8058_MOESM4_ESM.doc]
